# The Effects of Protein and Supplements on Sarcopenia in Human Clinical Studies: How Older Adults Should Consume Protein and Supplements

**DOI:** 10.4014/jmb.2210.10014

**Published:** 2022-10-31

**Authors:** Young Jin Jang

**Affiliations:** Major of Food Science and Technology, Seoul Women’s University, Seoul 01797, Republic of Korea

**Keywords:** Sarcopenia, protein, whey, amino acids, leucine, HMB

## Abstract

Sarcopenia is a condition in which muscle mass, strength, and performance decrease with age. It is associated with chronic diseases such as diabetes, cardiovascular disease, and hypertension, and contributes to an increase in mortality. Because managing sarcopenia is critical for maintaining good health and quality of life for the elderly, the condition has sparked concern among many researchers. To counteract sarcopenia, intake of protein is an important factor, while a lack of either protein or vitamin D is a major cause of sarcopenia. In addition, essential amino acids, leucine, β-hydroxy β-methylbutyrate (HMB), creatine, and citrulline are used as supplements for muscle health and are suggested as alternatives for controlling sarcopenia. There are many studies on such proteins and supplements, but it is necessary to actually organize the types, amounts, and methods by which proteins and supplements should be consumed to inhibit sarcopenia. In this study, the efficacy of proteins and supplements for controlling sarcopenia according to human clinical studies is summarized to provide suggestions about how the elderly may consume proteins, amino acids, and other supplements.

## Introduction

Sarcopenia is age-related loss of skeletal muscle mass and strength [1]. This disorder is associated with increased risk of falls and fractures, and it is also related to obesity and the development of insulin resistance, hypertension, diabetes, and cardiovascular disease [2]. The elderly with sarcopenia are more likely to have new depression symptoms than the non-sarcopenic elderly [3]. Several studies have shown that sarcopenia is associated with a significantly higher risk of mortality in adults and older adults [4, 5]. Moreover, risk of mortality was especially and directly related to strength in males older than 60 years. Rate of loss of strength was an important factor in mortality in those younger than 60 [6]. Therefore, sarcopenia was formally recognized as a disease and given the code ICD-10-CM (M62.84) in 2016 [7].

Various factors are involved in the onset and progression of sarcopenia. Lifestyle–related problems such as low physical activity, obesity, and smoking increase the risk of sarcopenia [8]. Nutrition factors such as low protein intake and calorie and vitamin D deficiency are also main causes of sarcopenia [8]. Chronic inflammation also affects sarcopenia, as pro-inflammatory cytokines such as interleukin-6 (IL-6), TNF-α, and C-reactive protein (CRP) increase adiposity and impair the protein synthesis pathway in skeletal muscles, which promotes the pathogenesis of sarcopenia [9]. Mitochondria dysfunction is associated with sarcopenia because its impairment results in increased reactive oxygen species generation and chronic inflammation [10]. Hormonal changes such as testosterone and estrogen can also influence muscle mass and strength [11].

Although various factors are involved in progression of sarcopenia, the increase of exercise and protein intake are modifiable life factors and a basic strategy to prevent sarcopenia [12]. The current recommended dietary allowance (RDA) of protein is 0.8 g/kg/day, but several studies suggest that higher protein consumption than the RDA is required to preserve muscle mass and function in the elderly [12]. There are concerns about the side effects of protein over intake, which include obesity, kidney disease, gout, and osteoporosis. Therefore, in this review, we summarized recent human clinical studies to understand the type, amount, and duration of protein intake and exercise required to control sarcopenia. The efficacy of amino acid-related supplements normally used in sports nutrition, such as branch-chain amino acid (BCAA), leucine, and β-hydroxy β-methylbutyrate (HMB), have also been discussed for preserving muscle mass and function in the elderly [12]. Moreover, several studies have suggested that consuming multiple combinations of protein, amino acids, and vitamins may be more effective than single protein intake [12]. The combination effect of protein and other supplements has also been described [12]. Research on proteins or supplements that can improve sarcopenia without exercise have been selected first. Studies examining the effect of combination with exercise are cases with protein or supplements that have no effect on improving sarcopenia without exercise, or that have reduced intake dose when combined with exercise.

## Protein and Sarcopenia

Studies have reported that protein supplementation did not improve lean body mass nor physical function in older adults. Whey and casein supplement (0.5 g/kg/day, total protein intake: 1.3 g/kg/day) in older adults for 6 months did not increase lean body mass, muscle performance, nor physical function [13]. In addition, 40 g of whey protein isolate supplementation (total protein intake: 1.5 g/kg/day) for 12 weeks, even with resistance exercise in healthy, elderly adults (60-80 years), did not enhance muscle strength and mass [14]. However, the positive effect of milk or whey protein supplementation was investigated in the elderly consuming a low amount of protein under 1.0 g/kg/day [15]. Several studies demonstrated that 1.2-1.5 g/kg/day of milk or whey protein isolate (WPI) with resistance exercise enhanced muscle mass, performance or function in older adults (Table 1). The renal function was not adversely affected up to 1.5 g/kg/day of high protein intake [16]. In case of plant-based protein, soy isolate protein is not less effective than a half dose of WPI [17]. Plant-based protein is generally of lower quality with a lower amino acid profile and reduced bioavailability [18]. Although animal-based protein such as milk protein or WPI is more effective than plant-based protein for overcoming sarcopenia, various sources of plant-based protein should be developed as protein sources for the same goal, as the numbers of vegetarian and environment-related issues continue to increase. Moreover, the effect of protein-based protein on muscle mass and function in the elderly should be investigated since several phytochemicals from plants have been demonstrated to have positive effects on muscle mass and function.

## Essential Amino Acids (EAAs)

Essential amino acids (EAAs) have been reported to increase muscle protein synthesis (MPS), and especially, a high proportion of leucine is important for optimal stimulation of MPS [22]. However, only a few studies have demonstrated the effect of EAA on muscle mass, strength, and function in older adults [23]. In addition, 8-15 g/day of EAA supplementation for 8 weeks to 18 months enhanced lean body mass or muscle function (Table 2). However, there are few results showing that EAA supplementation increases both muscle mass and function or strength in older adults. A high proportion of leucine in EAA is important for older adults. Also, 41% leucine in EAA is required for optimal stimulation of the rate of MPS in the elderly, although 21% leucine stimulates MPS in young adults [22]. EAA containing 40% leucine only increased both lean body mass and muscle function in the elderly, and EAA containing 20% leucine increased only muscle function [24].

## Branch-Chain Amino Acids (BCAAs)

Branch-chain amino acids (BCAAs), such as leucine, isoleucine, and valine, have a branched functional R group and belong to essential amino acid. BCAAs promote the anabolic pathway in muscle rather than the liver [25]. Leucine in particular is a potent activator of mTORC1 and stimulates muscle protein synthesis [26]. mTORC1 promotes protein synthesis and attenuates autophagy by regulating several downstream regulators, such as S6K1, 4E-BP1, and Ulk1 [26]. BCAAs before exercise increased intracellular BCAA levels during exercise and thus inhibit muscle protein breakdown [27]. Therefore, BCAAs are commonly used in sports nutrition. However, there are few studies of BCAA effect on sarcopenia [25]. A recent study showed that 12 g/day of BCAA for 6 months with 30 min exercise did not improve muscle mass in patients with cirrhosis having sarcopenia (protein intake: 1-1.2 g/kg/day) [28]. Supplementation of leucine only is also not promising for preventing sarcopenia [29]. Leucine supplementation (7.5 g/day) for 12 or 24 weeks did not alter lean mass, muscle strength, nor walking speed in healthy, elderly people or type 2 diabetic men [30, 31]. Leucine supplementation (10 g/day) with exercise for 13 weeks only increased walking speed without the increase of lean mass and muscle strength [29].

## β-Hydroxy β-Methylbutyrate (HMB)

β-Hydroxy β-methylbutyrate (HMB) is produced by leucine metabolism in the body and has been identified to attenuate sarcopenia by promoting protein synthesis pathway and suppressing proteolysis pathway [32]. Sixty grams daily of leucine is required to make 3 g of HMB to maximally promote muscle protein synthesis, but it is impossible to consume 60 g of leucine per day [32]. Therefore, HMB consumption is more effective than leucine and has been used as a nutritional supplements for athletes to improve muscle mass and performance [33]. Clinical studies of the effect of HMB supplementation on sarcopenia have been well documented recently [34]. In addition, 1.5 g of HMB supplementation during 8 weeks or 24 weeks without exercise improved muscle strength and quality in the elderly [35, 36]. Two grams of HMB supplementation for 2-4 weeks reduced muscle degradation in nursing home residents receiving tube feeding [37]. Meanwhile, 3 g of HMB supplementation for 8 weeks also prevented acute decline in muscle mass in older people with >10 days’ bed rest [38]. It was shown that a daily 2-3 g of HMB consumption is safe without any effect on lipid profile, biochemistry, hepatic, and renal failure [39].

## Creatine

Creatine is a nitrogenous organic acid found in red meat, seafood, and poultry and also produced endogenously at about 1 g/day [40, 41]. Approximately 95% of creatine mainly resides in skeletal muscle and a small amount (~5%) of creatine is located in the testes and brain [42]. About 66% of intramuscular creatine is phospho-creatine, and its hydrolysis releases a phosphate group for synthesizing ATP [40]. Therefore, creatine has been used to improve exercise performance in athletes [41]. Many studies regularly showed that creatine supplementation increases strength, lean mass, and muscle morphology with heavy resistance exercise more so than resistance exercise alone [41]. The supplementation of creatine in the elderly also showed a positive effect on muscle mass and performance. When creatine was supplemented to the elderly above 59 years of age without resistance exercise, the effect was shown on muscle mass and function at 0.3 g/kg/day, although a high dose of creatine at 4-20 g/day was also effective [43]. Furthermore, 0.08-0.1 g/kg/day of creatine supplementation with resistance exercise enhances lean body mass, leg density, and exercise performance, such as leg press and chest press strength, suggesting a small amount of creatine is required to overcome sarcopenia with exercise [44]. The study of creatine doses higher than 0.3 g/kg/day on sarcopenia was excluded.

## Citrulline

Citrulline is non-essential α-amino acid found in watermelon. It is metabolized to arginine, a key metabolite in nitric oxide synthesis and the urea cycle, and therefore it is recognized as the arginine precursor [45]. Arginine is an essential amino acid that plays an important role in nitric oxide production and the vasolidation process. Arginine is metabolized for excretion through the urea cycle or used for protein synthesis in the rest of tissues [45]. Ten grams of citrulline supplementation in malnourished elderly patients improved amino acid availability in both genders and increased lean mass, appendicular skeletal muscle mass, and decreased fat mass only in women [46]. Other studies also showed that 10 g of citrulline supplementation with high intensity exercise increased muscle strength and decreased fat mass in obese elderly patients compared to an exercise-only group [47]. The combination of citrulline and malate improved walking speed at 3 g, suggesting citrulline malate is an effective form of supplementation. Indeed, 0.18 g/kg citrulline for 7 days does not enhance protein synthesis in healthy people, but the combination of citrulline and malate (8 g) gives beneficial effects such as maximal strength, power, and number of repetitions performed to failure in female athletes [48].

## Fish Oil

Chronic low-grade inflammation is associated with aging and therefore involved in the development of sarcopenia [49]. Omega-3 fatty acid (ω-3) has been reported to attenuate sarcopenia due to its anti-inflammatory properties [49]. The effect of ω-3 supplementation on sarcopenia with or without exercise has been well documented recently [50]. Studies showing the positive effects on sarcopenia of low doses of ω-3 supplementation have been mentioned in this section. Supplementation of ω-3 with or without resistance exercise improved muscle strength or function in older adults but does not enhance muscle mass [50]. Also when combined with resistance exercise, even low intake of ω-3 was effective [51].

## Vitamin D

Most studies investigating the effect of vitamin D supplementation on sarcopenia have shown that vitamin D-only supplementation did not enhance muscle mass, strength or performance in older adults [52]. For example, approximately 2,000 IU of vitamin D did not reduce the risk of falling in older adults [53]. Also, 3,750 IU of vitamin D improved neither sarcopenia indicators nor adiposity in older adults [54]. High dose of vitamin D supplementation (40,000 IU) did not enhance muscle strength and mass compared to control group in postmenopausal women [55]. A limited number of studies demonstrated an increase in muscle strength in older adults with 25(OH)D ≤ 25 nmol/l [56]. One study showed that vitamin D (10,000 IU) supplementation increased skeletal muscle mass but not muscle strength in pre-sarcopenic older adults with vitamin D deficiency [57].

## Combination Effect of Protein and Other Supplements

Whey protein is rich in leucine and effective to counteract sarcopenia as we described in a previous section [58]. Leucine content of protein and essential amino acids are important factors to attenuate sarcopenia although leucine only did not improve sarcopenia factors in older adults [29]. Low vitamin D level is correlated with reduced muscle mass and impaired physical performance in the frail elderly although vitamin D supplementation is not effective on sarcopenia indicators [59]. Therefore, the combination effects of leucine and protein, and vitamin D have been examined. Vitamin D and leucine-rich whey protein enhances lean body mass and muscle function in sarcopenic older adults [60]. Ursolic acid is a phytochemical abundant in apple and has been reported to enhance muscle mass and function in various muscle atrophy animal models [61]. However, recent clinical studies showed that ursolic acid supplementation did not increase muscle strength, mass, serum IGF-1, and Akt-mTORC1 pathway in resistance-trained men [62, 63]. Therefore, the combination of various protein and supplements is a good strategy to counteract sarcopenia rather than one protein or supplement.

## Conclusion

As previously described in present study, the effects of protein and supplements on muscle mass and function differed between adults and the elderly. Although the positive effects of protein and supplements were found in adults and athletes, they may not be the same in the elderly. Therefore, further studies on various protein, supplement and pharmaceuticals in sarcopenia should be performed. The RDA of protein for elderly should be revised, and the standard of protein and supplement intake should be reestablished for the elderly. This review could be used as a protein intake guideline for the elderly to attenuate sarcopenia and healthy aging.

## Figures and Tables

**Table 1:** Studies showing positive effect of protein supplementation on sarcopenia.

Protein	Subject	Amount	Total protein intake	Duration	Exercise	Results	Ref.
Milk protein	Physically active older adults (67-73 years) who were training on a 4-day walking event of 30, 40, 50 km/day with low habitual protein intake (<1.0 g/kg/day)	31 g	1.29 ± 0.28 g/kg/day	12 weeks	-	Lean body mass ↑ Fat mass ↓	[15]
Whey protein	Prefrail and frail malnourished elderly aged 70-85 years (0.8 g/kg/day)	9.3 g whey protein, 0.5g fat, 0.2g cocoa powder	1.5 g/kg/day (1.2 g/kg/day: no significance)	12 weeks	-	Appendicular skeletal muscle mass, skeletal muscle mass index, gait speed ↑	[19]
Whey protein isolate (WPI)	Healthy older men (mean age 67 ±1)	25 g WPI (including~3g leucine) twice directly after breakfast and lunch	1.5 g/kg/day (breakfast 0.45, lunch 0.55, dinner 0.5 g/kg/day)	12 weeks	Resistance exercise (RE) twice /week	WPI alone: gait speed ↑ RE alone: muscle strength, fat free mass, physical function ↑ No synergistic effects WPI and RE	[16]
Milk protein	Mobility-limited older men and women (85±6 years)	34 g of milk protein (17 g protein x 2 times, morning, evening)	1.22 g/kg/day	10 weeks	Heavy-load strength training, three times a week	Leg lean mass, thickness, knee extensor strength, functional performance ↑	[20]
Milk protein	Physically active older men and women (≥65 years)	Total 36.8 g milk protein concentrate daily with 31 g protein, 1.1 g fat, 14.5 g lactose, consumed one during breakfast and one within 30 min after exercise	1.35 g/kg/day (Daily protein intake 0.92±0.27 g/kg/day)	12 weeks	Trained for 4 day walking event of 30, 40, 50 km/day	Lean body mass ↑ Fat mass ↓	[15]
Milk protein concentrate	Elderly >60 years	Milk protein drink (7.0 g of carbohydrate, 10.1 g of protein and 0.2 g of fat)	1.48 g/kg/day (Daily dietary protein intake is 1.2-1.3 g/kg/day)	6 months	Daily exercise training (low-tomoderate intensity)	Muscle mass ↑	[21]
Soy protein isolate (SPI) vs. whey protein isolate (WPI)	Elderly aged 71±5 years	WPI 20, 40 g SPI 20, 40 g	WPI, SPI 20 g: 1.28 g/kg/day, WPI 40 g: 1.52 g/kg/day, SPI 40 g: 1.55 g/kg/day (Daily protein intake 1.0 g/kg/day)	1 day	Rest or exercise Unilateral kneeextensor resistance exercise	Rest+WPI 20 g, 40 g, exercise+WPI 20 g, 40 g, exercise+SPI 40 g: myofibrillar protein fractional synthetic rate ↑ 40 g SPI was less effective than 20 g WPI	[17]

**Table 2:** The positive effects of well-known supplements on muscle mass and function in older adults.

Supplement	Subject	Amount	Duration	Exercise	Results	Reference
Essential amino acid (EAA)	Elderly patient (75-95 years) with sequelae of coronary artery disease (73%), femoral fracture (34%)	4 g x 2 time = 8 g/day (containing 31% leucine)	8 weeks	-	Quality of life, muscle function, diet profile ↑	[64]
	Healthy older women (68±2 years)	7.5 g x 2 times = 15 g/day (containing 18.6% leucine)	3 months	-	Lean body mass ↑	[65]
	Older adults with sarcopenia (66-84 years	8 g x 2 times =16 g/day (containing 31% leucine)	18 months	-	Le an body mass ↑	[66]
	Older adults (65-75 years)	7.5 g x 2 times =15 g/day (containing 20% or 40% leucine)	12 weeks	-	Functional performance, lean tissue mass ↑	[24]
Beta-hydroxybeta-methyl butyrate (HMB)	Bedridden or sedentary elderly	1.5-3 g	2-24 weeks	-	Muscle degradation ↓ Strength, function ↑	[34]
Creatine	Elderly women (59-90 year)	0.3 g/kg/day	7 days	-	Sit-to-stand, bench press and leg press, isometric knee extension and flexion, peak power, tandem gait ↑ fat free mass ↑	[43, 67]
	Elderly women (mean 65 years old)	0.08 g/kg/day	12 weeks	3 day/week	Fat free mass, muscle mass, bench press, knee extension, biceps curl ↑	[68]
	Elderly men and women (50-77 year)	0.1 g/kg/day	8 weeks-1 year	3 day/week	Leg press strength, lower body strength, muscle thickness, lean tissue mass, bench press, chest press, muscle density ↑	[44, 69]
Fish oil	Female, aged 65 years or older, sarcopenic according to the EWGSOP criteria	EPA 440 mg, DHA 220 mg	96 days	-	Muscle strength, performance ↑	[70]
	Elderly aged 71.2 years	Krill oil 4 g/day (772 mg EPA, 384 mg DHA)	6 months	-	Knee extensor maximal torque, grip strength, vastus lateralis muscle thickness ↑	[71]
	Female aged 60-76 years	5 g/day (2 g EPA, 1g DHA)	12 weeks	-	Lean mass, timed-upand-to test ↑	[72]
	Elderly aged 65 years	2 g/day (~0.4 g/d EPA, 0.3 g/d DHA)	90 days	Resistance exercise, 3 times/week for 12 weeks	Peak torque and rate of torque development, chair-rising performance ↑	[51]
Citrulline	Elderly aged 60-73 years	Citrulline-malate 3 g/day	6 weeks	-	Walking speed ↑	[45, 73]
	Malnourished older patients (80-92 years)	10 g	3 weeks	-	Amino acid availability, lean mass, appendicular skeletal muscle mass ↑ in women Fat mass ↓ in women	[46]
	Obese elderly (BMI 30-40 kg/m2, HIIT+CIT:67.2±5.0 years, HIIT+placebo: 68.1±4.1 years	10 g	12 weeks	High intensity interval training (HITT)	Adding citrulline to HIIT: muscle strength ↑ , fat mass ↓	[47]
Vitamin D	Pre-sarcopenic, deficient in vitamin D aged 73.31 years	10,000 IU of cholecalciferol	3 times a week for 6 months	-	Appendicular skeletal muscle mass ↑	[57]

**Table 3:** Combination effect of protein and other supplements.

Supplement	Subject	Amount	Exercise	Duration	Results	Reference
Whey protein, leucine, vitamin D	Older adults (≥65 years) having sarcopenia	12.8 g protein (including 8.5 g of whey protein concentrate), 1.2 g leucine, 120 IU vitamin D, Meal protein intake is 1.2-1.5 g/kg/day, equally distribute their meal time	-	12 weeks	Gait speed ↑	[74]
Whey protein, leucine, vitamin D	Older adults (≥65 years)	Twice daily (21 g whey, 3 g leucine, 800 IU vitamin D each serving) or once daily before breakfast	-	6-13 weeks	Appendicular lean mass ↑	[60, 75, 76]
Whey protein, leucine, vitamin D	Older adults (≥65 years)	Once daily (22 g whey, 4 g leucine and 100 IU of vitamin D each serving)	physical activity (20 min exercise/day, 5 times/week)	12 weeks	Fat free mass ↑ Handgrip strength ↑	[77]
Leucine-enriched protein (Casein, whey, soy) vitamin D, calcium	Healthy adult (50-80 years)	Protein 20 g (casein 50 %+ whey 40 %+ soy 10 %, total leucine 3000 mg), vitamin D 800 IU (20 μg), calcium 300 mg: twice daily (Total protein intake intervention group: ~1.5 g/kg/day, control group: 1.05±0.35 g/kg/day)	-	12 weeks	Lean body mass/body weight ↑	[78]
Essential amino acid, vitamin D, medium-chain triglyceride	Elderly nursing home resident (age of 86.6 years)	Essential amino acid 3g (containing L-leucine 1.2 g) and vitamin D (20 μg or 800 IU)-enriched supplement with medium-chain triglyceride 6 g	-	3 months	Grip strength walking speed, open-and close test performance, peak expiratory flow ↑	[79]
Whey, casein protein, ursolic acid, free BCAA, vitamin D	Older adults (>65 years) with (or at risk of) undernutrition	Casein 11 g, whey 11 g, free BCAA 7 g, ursolic acid 206 mg, vitamin D3 10.8 μg	-	12 weeks	Lean body mass, walking performance, mitochondrial function ↑	[80]
